# Risk factors for surgical site infection in women undergoing hysterectomy for gynecological cancers: a retrospective study

**DOI:** 10.3389/fmed.2026.1942311

**Published:** 2026-10-29

**Authors:** Lifei Tang, Miao Yang, Jie Hu

**Affiliations:** Department of Gynecology, The Affiliated People’s Hospital of Ningbo University, Ningbo, Zhejiang, China

**Keywords:** blood transfusion, diabetes mellitus, gynecological cancers, hysterectomy, surgical site infections

## Abstract

**Background:**

Hysterectomy is a common surgery performed in women. Most patients with gynecological cancers (GCs) undergo hysterectomy along with extensive resection and are prone to surgical site infections (SSIs). However, the risk factors for SSIs in this population remain unclear. This study focuses on this issue.

**Methods:**

Patients who underwent hysterectomy for GCs between January 2021 and December 2025 were selected from the electronic medical records of a single institution. The patients were classified into the non-SSI and SSI groups based on the occurrence of SSIs. Uni- and multivariate logistic regression analyses were used to evaluate risk factors among patient demographics and clinical characteristics.

**Results:**

In total, 558 patients were included in this study. The non-SSI and SSI groups included 468 and 90 patients, respectively. Univariate analysis indicated significant differences in six factors: diabetes mellitus (DM), intraoperative blood transfusion (IBT), intraoperative blood loss (IBL), surgical time, cervical cancer surgery, and ovarian cancer surgery (*P* < 0.05). Further multivariate logistic regression analysis showed that DM, IBT, IBL, surgical time, cervical cancer surgery, and ovarian cancer surgery were independent risk factors for SSIs. Receiver operating characteristic curve analysis of IBL and surgical time showed areas under the curve of 0.9420 and 0.8984, respectively.

**Conclusions:**

DM, IBT, greater IBL, extended surgical time, cervical cancer surgery, and ovarian cancer surgery were found to be risk factors for SSIs in patients undergoing hysterectomy for the treatment of GCs. The accurate identification of high-risk patients is critical to guide perioperative care and reduce SSI-related morbidity.

## 1 Introduction

Gynecological cancers (GCs), including ovarian, cervical, endometrial, vulvar, and vaginal cancers, have the second-highest incidence after breast cancer in women worldwide. In 2022, GCs accounted for 1,473,427 new cases and 680,372 deaths, with an incidence of 30.3/100,000 and a mortality rate of 13.2/100,000 (1, 2). Surgery is the most common intervention (3).

Hysterectomy is one of the most common surgeries performed in women, with the majority being performed for benign indications (4). Radical surgery for GCs, including hysterectomy, is characterized by extensive resection and significant surgical trauma (5). Procedures performed to treat GCs are associated with a significantly higher incidence of complications than those performed to treat benign gynecological diseases (6, 7). Surgical site infections (SSIs) are common complications of GC surgeries. SSIs are infections that occur at the incision site within 30 days of surgery or within 1 year when an implant is involved; they involve the skin, subcutaneous tissue, deep soft tissue, or any organ or cavity exposed during surgery. SSIs are the most common postoperative complications, constituting the majority of nosocomial infections and accounting for approximately 20%–38% of all hospital-acquired infections (8, 9). The SSI rate is approximately 0.5%–5% among all surgical patients, with an incidence of 5.7%–10% after major abdominal surgeries (10, 11). SSIs pose a significant public health threat, increasing morbidity and mortality, and imposing a heavy clinical and economic burden globally (12).

Patients undergoing hysterectomy are prone to SSIs (13), and many studies have focused on the risk factors for SSIs in patients undergoing hysterectomy (14–16). However, few have explored this issue in patients with GCs. We aimed to address this by investigating the prevalence and risk factors for SSIs in patients undergoing hysterectomy for GCs.

## 2 Methods

### 2.1 Study design and eligibility

This retrospective study included women who had undergone hysterectomies at the Affiliated People's Hospital of Ningbo University between January 2021 and December 2025. Electronic medical records and the hospital's active surveillance system for nosocomial infections were reviewed. The inclusion criteria were age ≥18 years, undergoing hysterectomy, and a diagnosis of GC. The exclusion criteria were secondary GC, preoperative gynecological infection at the time of surgery, and loss to follow-up within 30 days after surgery. All surgeries were performed by an experienced gynecologist. This study complied with the principles of the Declaration of Helsinki. The study protocol was approved by the Ethics Committee of the Affiliated People's Hospital of Ningbo University (NO. 2026-075).

### 2.2 Diagnosis of SSIs

SSIs were diagnosed when infection occurred within 30 days after the hysterectomy for GC and involved the following anatomical parts that were opened or manipulated: the skin or subcutaneous tissue at the incision site; deep soft tissues at the incision site; and the abdominal or pelvic cavities. Incisional SSIs were defined as infections involving the skin, subcutaneous tissue, or deeper muscle and fascial layers, whereas organ space SSIs referred to infection of any non-incisional anatomical structure involved in the surgery including intra-abdominal abscesses, anastomotic leaks or fistulas (17). Clinical findings included at least one of the following: purulent drainage; organisms isolated from an aseptically obtained culture of the drainage; fever (>38 °C), localized pain or tenderness; an abscess or other evidence of infection observed on direct examination, during reoperation, or by imaging examination; diagnosis of SSI by the surgeon or attending physician (18).

### 2.3 Perioperative care

A proposed bundle of care was implemented to reduce the incidence of SSIs; it included preoperative bathing or showering, intravenous prophylactic and postoperative antibiotics, skin preparation, intraoperative wound irrigation, intraoperative wound protection, good intraoperative hemostasis, surgical drain placement, and perioperative glycemic control (10, 19)

### 2.4 Postoperative observation

Postoperative surveillance for SSIs after hysterectomy for GCs included clinical assessments, laboratory tests, and imaging studies, when indicated. The initial postoperative wound inspection was performed after 48 h or sooner if wound exudate, local pain, or fever were present. Thereafter, it was performed on the day of suture removal or at discharge, when no earlier evaluation was indicated. Particular attention was paid to the presence of fever, persistent or worsening pelvic pain, and ileus. Laboratory monitoring included serial white blood cell (WBC) counts, and C-reactive protein and procalcitonin level measurements. However, an aberrant elevation or secondary increase in body temperature after postoperative day 3 prompted further investigation. Ultrasonography or contrast-enhanced computed tomography was used to confirm the diagnosis in patients with suspected deep or pelvic collections.

### 2.5 Follow-up regimen

Post-discharge surveillance for wound healing and other symptoms was performed via outpatient clinic visits or telephone interviews within the first 30 postoperative days. Surgical incisions were evaluated at each follow-up visit within 30 days after surgery. Patients were advised to present promptly for reevaluation if they developed fever, abdominal pain, or local signs of incisional inflammation, such as erythema, swelling, or tenderness. For tumor surveillance, follow-up evaluations were scheduled at 3-month intervals during the first 2 years, 6-month intervals from years 3–5, and annually thereafter in patients with stable disease.

### 2.6 Data collection

The following data were collected: (1) preoperative patient demographics: age, body mass index (BMI), smoking status, and alcohol intake; (2) clinical characteristics: Nutritional Risk Screening 2002 (NRS-2002) score, American Society of Anesthesiologists (ASA) score, WBC count, serum albumin level, blood creatinine level, history of diabetes mellitus (DM), hypertension, hypercholesterolemia, or hyperlipidemia, liver dysfunction, history of other abdominal surgery, intraoperative blood transfusion (IBT), intraoperative blood loss (IBL), surgical time, neoadjuvant chemotherapy, types of GC, surgical approach, concomitant non-gynecological organ resection, and Tumor-Node-Metastasis (TNM) classification stage.

### 2.7 Statistical analysis

All statistical evaluations were performed using SPSS software (version 27.0; SPSS Inc., Chicago, IL, USA). For between-group comparisons, continuous variables were analyzed using the independent samples Student's *t*-test, whereas categorical variables were compared using the Pearson chi-squared test or Fisher's exact test, depending on the expected cell frequency. To explore the predictors of SSIs, we first performed univariate exact logistic regression analyses. Candidate variables demonstrating a marginal association (defined as *P* < 0.10) were then included in a stepwise multivariate binary logistic regression model to identify the independent risk factors. The predictive accuracy and clinical utility of the resultant model were evaluated using receiver operating characteristic (ROC) curve analysis. A two-tailed *P* < 0.05 indicated statistical significance in all analyses.

## 3 Results

### 3.1 Baseline patient characteristics

In total, 558 patients who were diagnosed with GCs were included in this study. The mean age of the cohort was 58.5 ± 13.2 years. Based on the occurrence of SSI, the patients were stratified into two groups: the non-SSI group (*n* = 468) and the SSI group (*n* = 90). Fifty-five patients (61.1%) were diagnosed with incisional SSIs and thirty-five (38.9%) with organ-space SSIs. The overall study algorithm and analytical workflow are illustrated in Figure 1.

**Figure 1:**
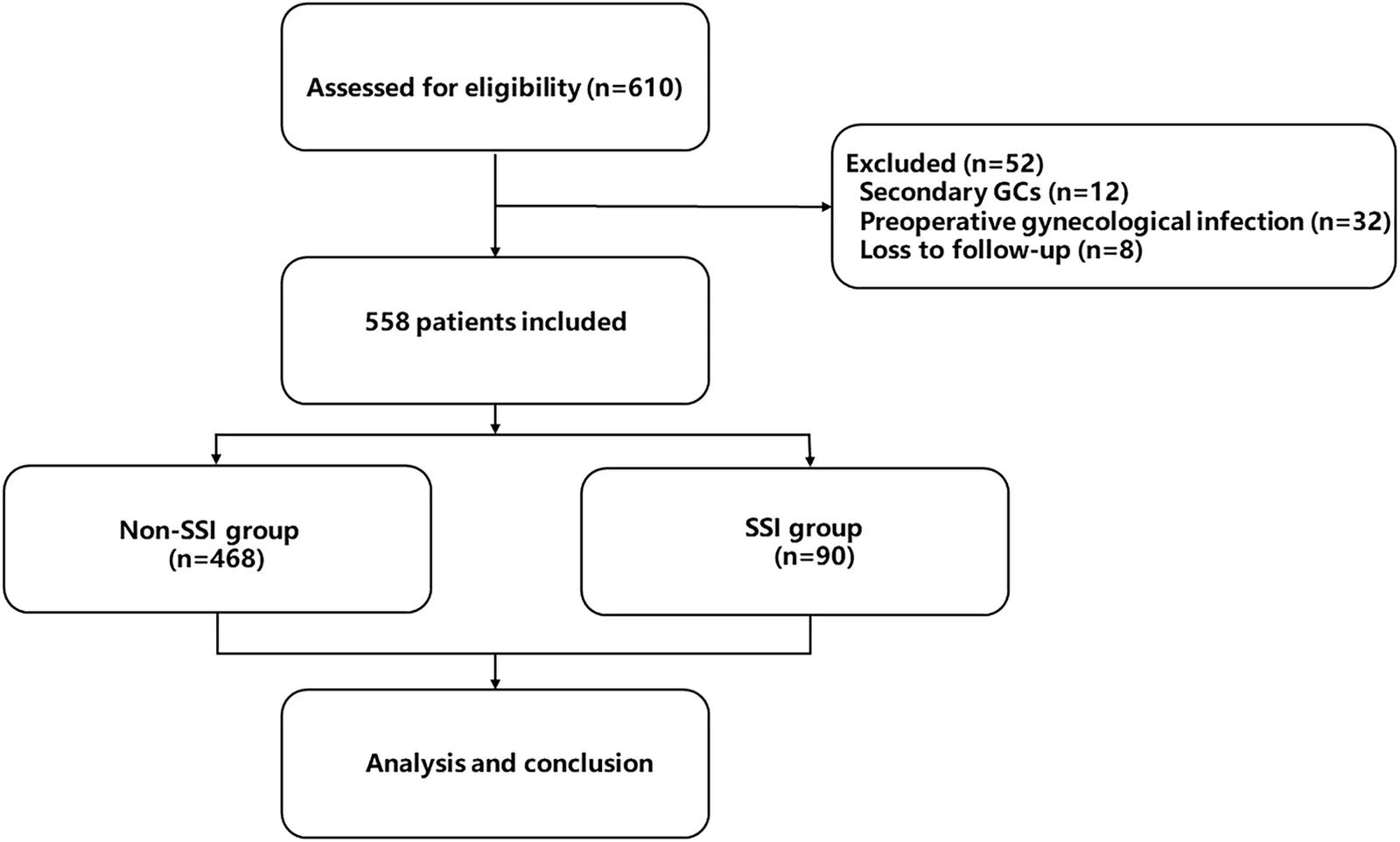
The flow chart of the study population.

The patient demographics and clinical characteristics of the two groups were compared. No significant differences were observed between the groups with respect to age, BMI, smoking, alcohol intake, NRS-2002 score, ASA score, WBC count, serum albumin level, serum creatinine level, history of hypertension, hypercholesterolemia, or hyperlipidemia, liver dysfunction, history of prior abdominal surgery, neoadjuvant chemotherapy, surgical approach, concomitant nongynecological organ resection, or TNM stage (*P* > 0.05). In contrast, significant intergroup differences were noted for DM, IBT, IBL, surgical time, and GC type (*P* < 0.05; Table 1).

**Table 1:** Comparison of baseline demographics and clinical variables between the two groups.

Variables	Non-SSI group (*n* = 468)	SSI group(*n* = 90)	*P* value
Age (years)	58.5 ± 13.5	58.6 ± 11.7	0.956
BMI (kg/m2)	23.8 ± 2.4	23.6 ± 2.4	0.217
Smoking, *n* (%)			0.310
Yes	8 (1.7%)	3 (3.3%)	
No	460 (98.3%)	87 (96.7%)	
Alcohol intake, *n* (%)			0.156
Yes	20 (4.3%)	7 (7.8%)	
No	448 (95.7%)	83 (92.2%)	
NRS-2002 score			0.372
< 3	378 (80.8%)	69 (76.7%)	
≥ 3	90 (19.2%)	21 (23.3%)	
ASA score			0.763
I	144 (30.8%)	27 (30.0%)	
II	288 (61.5%)	54 (60.0%)	
III	36 (7.7%)	9 (10.0%)	
WBC counts (×10^9^/L)	6.1 ± 1.9	6.7 ± 3.0	0.088
Albumin (g/L)	40.1 ± 4.1	41.0 ± 6.7	0.051
Creatinine (*μ*mol/L)	57.8 ± 13.3	58.0 ± 7.4	0.810
DM, *n* (%)			0.005
Yes	81 (17.3%)	27 (30.0%)	
No	387 (82.7%)	63 (70.0%)	
Hypertension, *n* (%)			0.098
Yes	180 (38.5%)	43 (47.8%)	
No	288 (61.5%)	47 (52.2%)	
Hypercholesterolemia or hyperlipidemia, *n* (%)			0.065
Yes	99 (21.2%)	27 (30.0%)	
No	369 (78.8%)	63 (70.0%)	
Liver dysfunction, *n* (%)			0.859
Yes	44 (9.4%)	9 (10.0%)	
No	424 (90.6%)	81 (90.0%)	
Prior abdominal surgery history, *n* (%)			0.058
Yes	85 (18.2%)	9 (10.0%)	
No	383 (81.8%)	81 (90.0%)	
IBT, *n* (%)			< 0.001
Yes	63 (13.5%)	54 (60.0%)	
No	405 (86.5%)	36 (40.0%)	
IBL (mL)	116.2 ± 73.2	309.7 ± 144.3	< 0.001
Surgical time (minutes)	173.7 ± 59.0	312.4 ± 90.0	< 0.001
Neoadjuvant chemotherapy, *n* (%)			0.185
Yes	9 (1.9%)	18 (20.0%)	
No	459 (98.1%)	72 (80.0%)	
GC type, *n* (%)			< 0.001
Endometrial cancer	211 (45.1%)	10 (11.1%)	
Cervical cancer	135 (28.8%)	40 (44.4%)	
Ovarian cancer	117 (25.0%)	39 (43.4%)	
Vaginal cancer	5 (1.1%)	1 (1.1%)	
Surgical approach, *n* (%)			0.163
Open	207 (44.2%)	47 (52.2%)	
Laparoscopic	261 (55.8%)	43 (47.8%)	
Concomitant non-gynecologic organ resection, *n* (%)			0.398
Yes	9 (1.9%)	3 (3.3%)	
No	459 (98.1%)	87 (96.7%)	
TNM stage, *n* (%)			0.316
I	342 (73.1%)	63 (70.0%）	
II	81 (17.3%)	14 (15.6%)	
III	27 (5.8%)	15 (11.1%)	
IV	18 (3.8%)	3 (3.3%)	

SSI, surgical site infections; BMI, body mass index; NRS-2002, nutritional risk screening 2002; ASA score, American Society of Anesthesiologist score; WBC, white blood cell; DM, diabetes mellitus; IBT, intraoperative blood transfusion; IBL, intraoperative blood loss; GC, gynecological cancer; TNM, tumor-node-metastasis.

### 3.2 Risk factors for SSIs in hysterectomy for GCS

ROC curve analysis was performed for IBL and surgical time, yielding areas under the curve (AUC) of 0.9420 and 0.8984, respectively (Figure 2). Variables with *P* < 0.10 in the univariate analysis were subsequently entered into a multivariable logistic regression model. This analysis confirmed six independent risk factors for SSIs after hysterectomy for GCs: DM [odds ratio [OR] 3.311, 95% confidence interval [CI] 1.325–8.272, *P* = 0.010], IBT (OR 4.083, 95% CI 1.762–9.461, *P* = 0.001), higher IBL (OR 1.012, 95% CI 1.008–1.016, *P* < 0.001), longer surgical time (OR 1.020, 95% CI 1.014–1.027, *P* < 0.001), cervical cancer surgery (OR 7.692, 95% CI 2.488–23.810, *P* < 0.001), and ovarian cancer surgery (OR 6.250, 95% CI 1.953–20.000, *P* = 0.002) (Figure 3).

**Figure 2:**
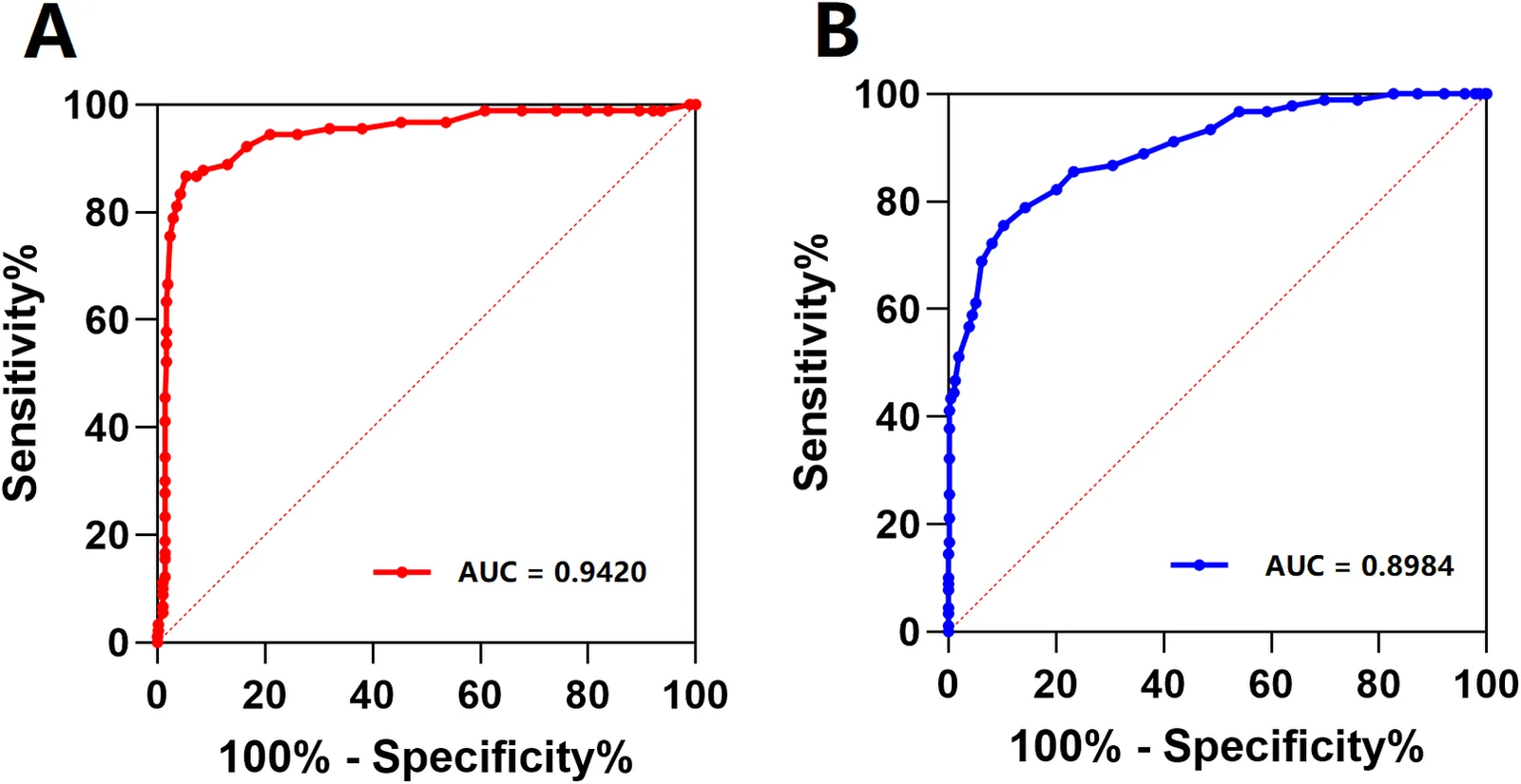
ROC curves of the logistic model. ROC, receiver operating characteristic. **(A)** intraoperative blood loss; **(B)** surgical time.

**Figure 3:**
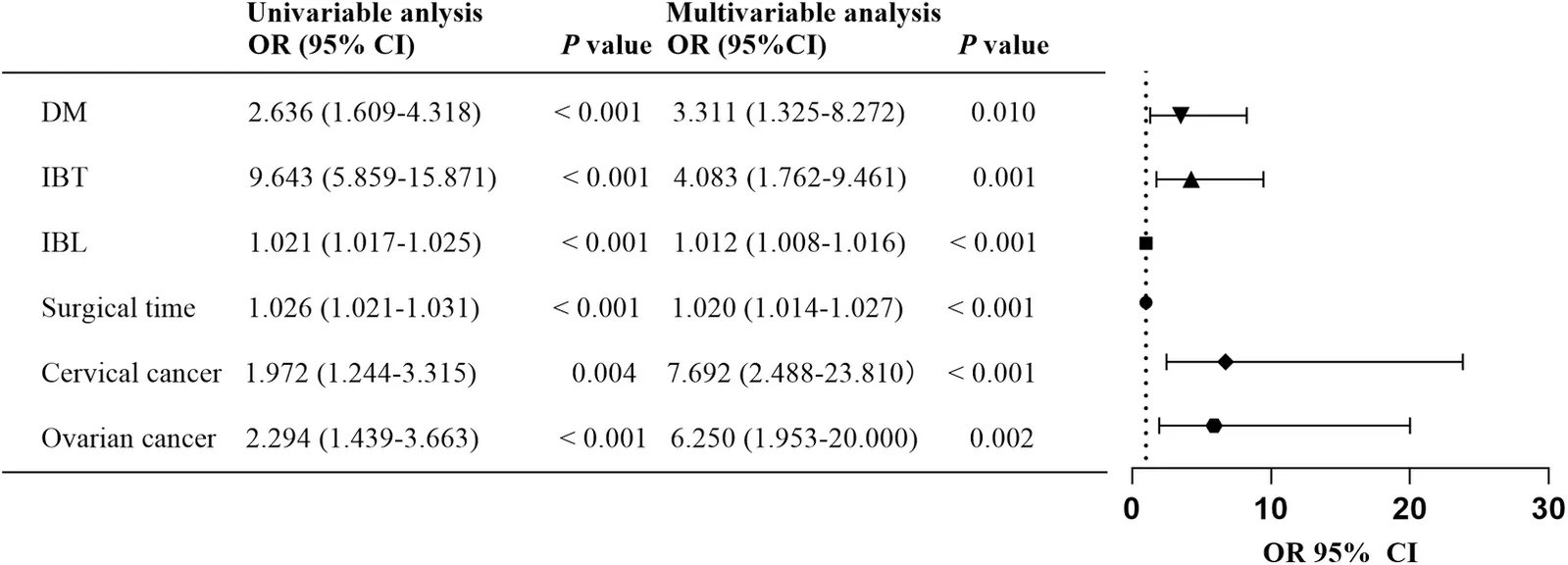
Forest plot of the results of the uni- and multivariable regression analyses identifying risk factors for surgical site infections after hysterectomy for gynecological cancers. Univariable OR and 95%CI were first calculated, followed by multivariable adjustment to screen for independent predictors. DM, diabetes mellitus; IBT, intraoperative blood transfusion; IBL, intraoperative blood loss.

## 4 Discussion

Infection is the second most common cause of death in obstetric and gynecological patients after postpartum hemorrhage (20). Hysterectomy is one of the most frequently performed non-obstetric gynecological surgeries in western countries (21). Abdominal surgery is particularly prone to SSIs because of the anatomical complexity of the abdominal cavity and its vulnerability to microbial contamination (22). Although surgical techniques for hysterectomy have evolved considerably from conventional open surgery to laparoscopic and robot-assisted approaches, postoperative SSIs remain an inevitable concern. SSIs are a major public health challenge worldwide. In patients with GCs, the immune system is often impaired due to the direct immunosuppressive effect of the tumor and aggressive interventions, such as surgery, chemotherapy, and radiotherapy (14). The incidence of SSIs ranges widely from 2%–21% after hysterectomy and is approximately twice as high in patients with GCs (15). SSIs are the most common healthcare-associated infections and are associated with extended hospital stays; they lead to readmission, substantial morbidity, increased mortality, and increased healthcare-related expenses (23). Therefore, accurate recognition of high-risk patients plays a vital role in directing perioperative management and lowering the morbidity associated with SSIs.

In accordance with the existing literature (24, 25), our findings identified DM as an independent risk factor for SSIs in hysterectomies performed for GC. In patients with DM, hyperglycemia impedes wound healing through multiple mechanisms: it reduces tissue perfusion and oxygenation, triggers endothelial dysfunction, and sustains a prolonged inflammatory state. It also suppresses neutrophil phagocytic function, decreasing bacterial elimination and increasing susceptibility to infection (26).

This study demonstrated that greater IBL is a risk factor for SSIs in women undergoing hysterectomy for GCs. Substantial IBL diminishes tissue perfusion and precipitates local ischemia at the surgical site. This state of hypoperfusion compromises innate immune activity and the wound healing cascade, which collectively heighten the postoperative infection risk.

We also observed a higher risk of SSIs among patients who underwent IBT, which is consistent with previous studies (27, 28). In addition to extensive IBL, severe abnormal uterine bleeding and cancer-related anemia are important reasons for blood transfusions in this patient population. The higher incidence of SSIs among patients with anemia is probably driven by inadequate tissue oxygenation, secondary to the reduced oxygen-carrying capacity associated with low hemoglobin levels. IBL contributes to SSIs through blood transfusion-related immunomodulation and creates a local hematoma environment that favors bacterial growth (9, 29, 30).

We found that GC surgeries with longer surgical times were associated with a greater risk of SSI than shorter procedures. Extended operative time is associated with an increased risk of bacterial contamination due to the protracted exposure of incisions to the operating room environment and resultant tissue dehydration, which can impair the skin barrier and predispose the wound to microbial invasion (31). Furthermore, the effective concentration of prophylactic antibiotics declines over time, necessitating intraoperative redosing during prolonged procedures. Previous studies have identified intraoperative antibiotic redosing as a key area for quality improvement (23, 32). However, this preventive antibiotic protocol was not implemented in a significant proportion of our patient cohort. Operative time is correlated with the complexity of the case and the surgical team's skill, proficiency, and experience using the adopted surgical approach. Most earlier studies on hysterectomy have reported that operative time tends to be longer for GCs than for benign gynecological conditions (33, 34). Lymph node dissection, which involves more extensive surgical wounds and greater soft tissue damage, increases surgical trauma and IBL. Morgan et al. (35) reported that the incidence of SSI was nearly twice as high in patients with cancer, which underscores the importance of optimizing surgical techniques and strategies to shorten the operative time and reduce the risk of SSI in this patient population.

Gynecological malignancy has been well established as a risk factor for SSI (15, 16, 36). Our study further compared the incidence of SSI across different types of GCs. In this cohort, cervical cancer surgery, which is a clean-contaminated procedure, was associated with a greater risk of SSIs. This rationale is likely attributable to the dense and pathogenic vaginal flora environment, where recurrent tumor bleeding and necrosis may increase the risk of intraoperative wound contamination. Moreover, the creation and closure of the vaginal cuff leaves a suture line in a nonsterile environment. Interestingly, ovarian cancer surgery was also identified as a predictor of SSIs in the current cohort. The underlying mechanisms may be that the procedure is generally lengthy, involves extensive tissue dissection, and is more traumatic. Increased postoperative bleeding and fluid collection are associated with an elevated risk of SSIs.

Our findings have several clinical implications. First, women with cervical or ovarian cancer should be counseled preoperatively and monitored closely after surgery, with particular attention to wound care. Second, strict perioperative glycemic control should be emphasized in diabetic patients, including preoperative glycated hemoglobin optimization and intraoperative and postoperative glucose monitoring. Third, strategies aimed at reducing IBL and avoiding unnecessary transfusion, such as meticulous hemostasis, correction of preoperative anemia, and adherence to restrictive transfusion thresholds, may help lower SSI risk. Finally, these risk factors may be used to stratify patients and target perioperative interventions toward those with the highest risk.

This study had several limitations. First, it was a single-center retrospective investigation, and our findings may not be fully representative of the broader patient population. Therefore, the generalizability of our results remains uncertain. Second, the retrospective nature of the study inherently subjects our data to potential selection bias, as we relied on existing medical records rather than prospectively collected data. Third, we were unable to control for all potential confounding variables, including lifestyle factors, socioeconomic status, and detailed medication histories, owing to database constraints. Fourth, not every patient in this cohort had obtainable drainage for bacterial culture, which may have limited the completeness of SSIs diagnosis. Finally, our clinical practices and treatment protocols may differ from those used in other settings. This further limits the external validity. Despite these limitations, our study provides valuable real-world evidence and highlights the need for prospective multicenter studies to validate our findings.

## 5 Conclusion

DM, IBT, greater IBL, longer surgical time, cervical cancer, and ovarian cancer were identified as independent risk factors for SSIs after hysterectomies for GCs. Although our findings warrant cautious interpretation due to the study limitations, the identification of high-risk patients remains critical for improving perioperative care and reducing SSI-related morbidity.

## Data Availability

The raw data supporting the conclusions of this article will be made available by the authors, without undue reservation.
